# Recent Advances in Bacterial Detection Using Surface-Enhanced Raman Scattering

**DOI:** 10.3390/bios14080375

**Published:** 2024-08-01

**Authors:** Manal Hassan, Yiping Zhao, Susu M. Zughaier

**Affiliations:** 1 College of Medicine, QU Health, Qatar University, Doha P.O. Box 2713, Qatar; mh2110611@qu.edu.qa; 2 Department of Physics and Astronomy, University of Georgia, Athens, GA 30602, USA; zhaoy@uga.edu

**Keywords:** surface-enhanced Raman scattering, pathogenic bacteria, high sensitivity, artificial intelligence

## Abstract

Rapid identification of microorganisms with a high sensitivity and selectivity is of great interest in many fields, primarily in clinical diagnosis, environmental monitoring, and the food industry. For over the past decades, a surface-enhanced Raman scattering (SERS)-based detection platform has been extensively used for bacterial detection, and the effort has been extended to clinical, environmental, and food samples. In contrast to other approaches, such as enzyme-linked immunosorbent assays and polymerase chain reaction, SERS exhibits outstanding advantages of rapid detection, being culture-free, low cost, high sensitivity, and lack of water interference. This review aims to cover the development of SERS-based methods for bacterial detection with an emphasis on the source of the signal, techniques used to improve the limit of detection and specificity, and the application of SERS in high-throughput settings and complex samples. The challenges and advancements with the implementation of artificial intelligence (AI) are also discussed.

## 1. Introduction

Bacteria are widespread and mostly nonpathogenic. However, pathogenic bacteria can cause various infectious diseases such as *Escherichia coli* (urinary tract infection (UTI), sepsis), *Salmonella* (food poisoning), *Neisseria gonorrhoeae* (sexually transmitted infection), *Neisseria meningitidis* (meningitis), *Staphylococcus aureus* (boils, cellulitis, abscesses, wound infections, toxic shock syndrome, pneumonia, sepsis, and food poisoning), and *Streptococcus* spp. (pneumonia, meningitis, ear infections, and pharyngitis) are a major clinical concern. To prevent complications and transmission, an early and sensitive detection strategy is pivotal [1]. Conventional tests such as Gram staining, culture-based methods, and biochemical assays are inexpensive, but the limitations involved are time-consuming detection procedures, expertise, and often limited sensitivity. Pre-enrichment of bacteria from a sample using a non-selective or selective broth culture for over 12 h is a strategy to increase bacterial load to facilitate detectable levels. For selective isolation and differentiation of associated Gram-positive (G+) and Gram-negative (G−) bacteria, a variety of chromogenic and fluorogenic culture media have been developed; however, these methods also suffer from longer detection times with generally limited sensitivity for discretion of bacterial strains. Techniques based on the advances in molecular biotechnology are also employed for rapid, real-time, sensitive, and specific pathogen detection, such as nucleic acid amplification tests and real-time polymerase chain reaction (RT-PCR) [2]. Recent methods for rapid detection of bacteria include immunological assays such as enzyme-linked immunosorbent assays (ELISAs); nucleic acid-based methods such as polymerase chain reaction (PCR), RT-PCR, loop-mediated isothermal amplification (LAMP); mass spectrometry-based matrix-assisted laser desorption ionization-time-of-flight MALDITOF; and analytical devices such as biosensors, most of which require expertise and have expensive, bulky equipment even though they provide accurate results, limiting their practical adoption [3].

**Figure 1:**
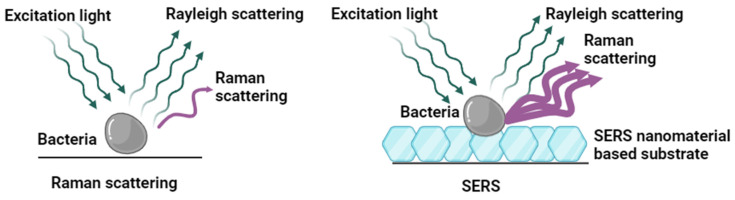
The comparison of normal Raman scattering and SERS scattering. (**Left panel**), Raman scattering of bacteria without surface enhancement. (**Right panel**), SERS enhancement by nanomaterials. Created with BioRender.com (accessed on 19 July 2024).

In 1928, C.V. Raman and K.S. Krishnan observed Raman scattering, where the inelastic scattering called stokes and anti-stokes was caused by the interaction of molecular vibration and incident light. The spectral peaks were sensitive to different vibrational modes of the molecular bonds. Hence, it gives unique “fingerprint” Raman peaks of the molecule, which can be used to determine the molecules [4,5]. There are several distinct forms of Raman spectroscopy, such as stimulated Raman, resonance Raman, transmission Raman, polarized Raman, and surface-enhanced Raman spectroscopy (SERS). The studies on the Raman effect underwent drastic developments since the 1970s with the accidental observation of enhanced scattering from a roughened metal surface due to the electromagnetic and chemical enhancements revealed later, that is, SERS [5]. SERS helped to overcome relatively poor sensitivity caused by the weak Raman scattering cross-section, which restricted the use of Raman spectroscopy for trace analysis [6,7]. Figure 1 provides a simple comparison between the normal Raman scattering and SERS scattering. The molecules adsorbed on proper nanostructures can induce enhancement as high as 14 to 15 orders of magnitude in comparison to normal Raman, making the technique sensitive enough to detect even a single molecule at low concentrations [8,9,10,11,12,13]. Typically, SERS employs specific metal nanoparticles, such as silver (Ag) and gold (Au), due to their plasmonic properties, which significantly boost sensitivity. This makes SERS a powerful platform for the rapid and sensitive detection of bacteria [14,15]. Other substrates used for SERS apart from metallic nanostructures are (1) 2D materials such as graphene/graphene oxide [16] and transition metal dichalcogenides (TMD) [17], (2) semiconductor nanostructures such as titanium dioxide [18], (3) composite materials such as metal-polymer composites [19], and (4) biological materials such as cellulose-based materials [20]. In 1998, the first SERS spectra of bacteria such as *Escherichia coli* were reported with spectra features attributed to the cell wall adsorbed on colloidal nano-silver particles [21]. Since then, the application of SERS in bacterial studies has expanded across various fields, including medical, environmental, and industrial microbiology, microbial systems biology, biological warfare countermeasures, and bioprocess monitoring [8]. These studies demonstrate that SERS can detect as few as a single bacterial cell or spore, making it a valuable point-of-care tool [21]. Over the past decades, SERS-based bacterial detection has primarily focused on understanding the origins and characteristics of the SERS signal of bacteria [22,23,24], creating substrates with high sensitivity and specificity to enhance bacterial detection [25,26,27,28], integrating SERS with conventional sample processing methods to improve detection efficiency [22], and applying SERS techniques to detect bacteria in complex, real-world samples [29]. The review outlines the development of SERS-based methods for microorganisms, focusing on the origins of bacterial SERS signals, techniques for improving sensitivity and specificity, and the application of SERS in high-throughput settings and complex samples. Additionally, it emphasizes advancements in the field facilitated by artificial intelligence.

## 2. Overview of Bacterial SERS Detection and Analytes

### 2.1. Different SERS Detection Methods: Label-Free and Label-Based

**Figure 2:**
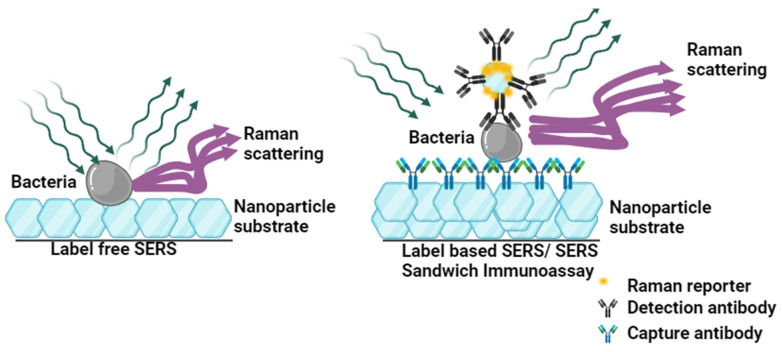
Illustrates the difference between Label-free and Label-based SERS. Created with BioRender.com (accessed on 19 July 2024).

Traditional plate culture-based methods, while accurate, require at least 18 h to confirm the bacterial presence, leading to delays in treatment, especially in cases of antibiotic resistance, as well as in prevention and management. In contrast, SERS has demonstrated extraordinary potential for bacterial detection due to its high enhancement and specificity. SERS-based techniques offer both label-free (direct) and label-based (indirect) detection strategies [30]. Label-free detection is a straightforward and accessible method that generates spectra based on the entire bacterial cell without using any additional Raman-active labels or reporters. The process involves directly loading the bacterial sample onto a SERS-active substrate (Figure 2). This method is advantageous because it is simpler and less time-consuming, providing direct detection of the sample without the need for complex preparation steps. However, it may have lower sensitivity and specificity compared to label-based methods, and the spectra can be more complex to interpret due to the presence of signals from various bacterial components. Label-based detection involves the use of Raman-active labels or reporters that are attached to or interact with bacterial cells. These labels enhance the Raman signal, allowing for highly sensitive and specific detection. The method typically involves preparing SERS substrates with metal nanoparticles functionalized with Raman-active molecules and targeting agents such as antibodies [31] or aptamers [32] that specifically bind to bacteria. This approach enhances sensitivity and specificity, making it possible to detect even low concentrations of bacteria. It also allows for multiplexing, where different Raman-active labels can be used simultaneously to detect multiple bacterial species or strains in a single sample. However, label-based detection is more complex and time-consuming due to the additional steps of functionalizing the substrates and preparing the labels. Both detection methods can benefit from additional performance enhancements through sample concentration techniques. Methods such as filtration, magnetic separation, and centrifugation are commonly employed to concentrate the bacterial sample, increasing the number of bacterial cells available for detection, thereby improving the detection sensitivity. Furthermore, surface modifications of conjugation molecules such as antibodies, aptamers, and peptides [33] to specifically capture target bacteria have been incorporated in detection to improve the specificity of the detection [34]. In summary, focusing on label-free SERS for bacterial detection offers numerous advantages, including simplified workflows, immediate results, and potential cost savings by eliminating the need for labeling agents. These methods also contribute to advancing our understanding of bacterial physiology and behavior, offering detailed chemical insights into structural components and metabolic activities. Embracing label-free SERS aligns with the goals of enhancing diagnostic accuracy and sensitivity, making significant strides in biosensing and microbiological research. Table 1 summarized different bacterial detections by SERS.

### 2.2. Target Analytes for SERS Bacterial Detection

Bacteria and their environment form an ecosystem, and the presence of bacteria in a sample is detectable either using chemical components intrinsic to bacterial cells or chemicals produced during the bacteria’s reproduction or interaction with the environment, which we call target analytes. The key features of target analytes for bacterial detection include specificity to the bacterial species or strain, surface accessibility for direct interaction with SERS substrates, and strong Raman activity for generating distinct signals. High binding affinity between analytes and SERS substrates or conjugation molecules, such as antibodies or aptamers, ensures effective capture and signal enhancement. Reproducibility of Raman signals across different samples is crucial for reliable identification and quantification, while minimal interference from other sample components reduces background noise and improves accuracy. Additionally, detecting target analytes at low concentrations is essential for high sensitivity, particularly in applications like early-stage infection detection or contamination monitoring. These features collectively contribute to the effectiveness and accuracy of SERS-based bacterial detection methods.

A diverse array of biomolecules and cellular components that contribute to distinctive signals crucial for identifying and characterizing bacteria are available for bacterial SERS detection. Key among these are bacterial biomarkers, which encompass chemicals produced by bacterial activities. Lipopolysaccharides (LPS), notably found in Gram-negative bacteria, are pivotal analytes in SERS [79,80]. Lipid A, core oligosaccharide, and O-specific polysaccharide chains are the components of LPS that are targeted for their immunogenic and pathogenic properties, providing valuable insights into bacterial identity and virulence. Bacterial metabolites also play a vital role in SERS detection [81]. These metabolites include small molecules such as amino acids, sugars, and organic acids, which reflect bacterial metabolic activity. Their detections not only inform on bacterial viability and physiological state but also aid in understanding microbial behavior under various conditions.

Peptidoglycan, a major constituent of the bacterial cell wall composed of sugars and amino acids, is prominently targeted in SERS due to its abundance and structural significance in maintaining cell integrity [82]. Proteins within bacterial cells, including structural proteins, enzymes, and membrane-associated proteins, serve as significant analytes [83]. Specific protein markers can differentiate between bacterial species or strains, providing crucial insights into bacterial physiology and function. Outer membrane proteins, exposed on the surface of bacterial membranes, are particularly valuable targets for understanding bacterial interactions with their environment and host cells, contributing to insights into bacterial behavior and pathogenicity. Moreover, nucleic acids such as DNA and RNA are targeted in SERS to identify bacterial genetic material [84]. Amplifying specific sequences or regions of bacterial DNA/RNA enhances the specificity and accuracy of SERS-based detection methods, enabling precise genetic identification and characterization of bacterial strains.

Overall, these analytes collectively bolster the robustness and versatility of SERS in identifying and studying bacterial cells across diverse applications in medical diagnostics, environmental monitoring, and microbiological research. Their targeted detection provides essential information for addressing challenges ranging from infection control to environmental protection and biotechnological advancements.

## 3. Label-Free Bacterial SERS Detection

### 3.1. SERS-Based Bacterial Gene Probe

The genetic material, DNA or RNA, of bacteria exhibits unique sequences specific to different bacterial species, serotypes, and strains, making it essential for PCR-based diagnostics. In Surface-Enhanced Raman Scattering (SERS), however, the nucleotide bases within DNA/RNA structures are structurally similar across bacterial types, leading to highly comparable SERS spectra. Minor variations in sequencing and length of DNA/RNA can induce subtle spectral changes, which are exploited to distinguish between different genetic targets. To enhance specificity, hybridization techniques are employed in DNA/RNA-based detection. This involves the precise pairing of complementary nucleic acid strands, facilitated by designed probes that match specific genetic sequences. Such methods are crucial for identifying pathogens, genetic mutations, or specific genes in biological samples with high sensitivity. Notably, in SERS, the small size of DNA/RNA molecules, measured in nanometers, allows them to efficiently interact with SERS hotspots, resulting in significantly amplified SERS signals compared to whole-cell detection methods. This capability underscores the potential of hybridization-based SERS assays in advancing molecular diagnostics and research applications [56,84,85].

The use of SERS for DNA detection dates to 1994, marked by the pioneering development of DNA gene probes that bind via hybridization to target DNA sequences [86]. Over the years, various SERS-based assays have evolved to detect bacterial genomes. Techniques such as the use of magnetic nanoparticles (MNPs) for DNA separation have been instrumental [87]. Recent research reported the detection of bacterial DNA using SERS. After the target DNA was bound, a short synthetic ssDNA that has been dye-modified and hybridized was used as the label. Specifically, three distinct PCR products tagged with three different dyes were simultaneously detected to display SERS’s multiplexing power. A separation-free SERS assay, which increases the SERS signal in the presence of target DNA [88], was also effective. After being amplified by PCR, specific *Staphylococcus epidermidis* bacterial DNA was found using SERS.

Multiplexed DNA detection of bacteria through a gold particle-on-wire system [61] was demonstrated. The system has an Au nanowire, on which the probe DNA is immobilized. Once the target DNA is hybridized to probe DNA, another Au nanoparticle-linked reporter probe, which is also complementary to the target DNA, is then hybridized. Hence, with the SERS, the seven isolates could be identified from the PCR products with a potential for multiplexing detection (2 isolates of *Enterococcus faecium*, 2 isolates of *Staphylococcus aureus*, 2 isolates of *Stenotrophomonas maltophilia*, and 1 isolate of *Vibrio vulnificus*). As a result, we can now identify rapidly without using PCR on direct samples with the development of SERS.

### 3.2. Biomarker-Based Detection

**Figure 3:**
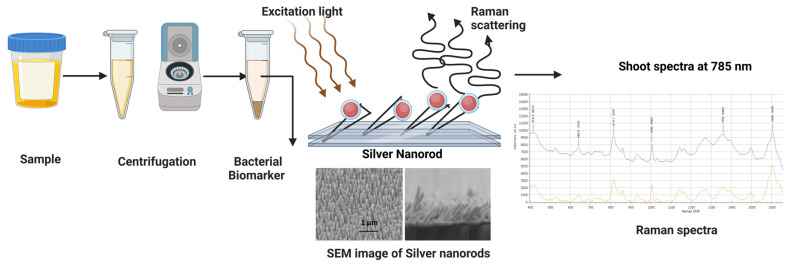
The workflow of SERS spectra collection. Created with BioRender.com (accessed on 19 July 2024).

The cell envelope that shares structural and chemical similarities with most bacterial species has been suggested as a source of bacterial Raman signal [89,90,91,92]. Therefore, without secondary labeling, it is difficult with Raman spectroscopy to classify closely related species, particularly from a mixture of bacteria. Hence, cells must be rinsed repeatedly and resuspended in water to eliminate any possible matrix or buffer residues. This process may induce cell rupture because of a disrupted osmotic balance, and the reproducibility of the assay may be impaired due to an unreliable sample constitution. These factors make it difficult to detect entire bacterial cells with Raman spectroscopy for clinical diagnosis [93]. Therefore, another approach involves indirectly detecting the bacteria by identifying the biomarkers that are released by the organism in the matrix fluids. These bacterial biomarkers can be shed during an infection in the host or be present in the cell wall. They can be in various forms, such as enterotoxins/endotoxins, cell surface markers such as LPS, teichoic acid, surface proteins, metabolites, and genetic markers. Silver film over nanospheres (AgFON) substrates were used to obtain the SERS spectra of a biomarker for spores of bacillus called calcium dipicolinate (CaDPA) and obtained a limit of detection (LOD) of 2.6 × 10^3^ spores of *Bacillus* [94]. Additionally, AuNPs were used to acquire the DPA SERS spectra that were recovered from *Bacillus* spores [72] and silver colloids [95]. Pyocyanin (PCN) can be used as a major biomarker for the detection of *Pseudomonas aeruginosa* [75]. Pyocyanin, a secondary metabolite produced by *Pseudomonas aeruginosa*, can be quantified up to five orders of magnitude with a limit of quantification of 1 ng/mL. SERS of PCN were obtained using Ag NR substrates with a very low limit of detection, 2.38 × 10^−8^ mol/L in both aqueous solutions and spiked clinical sputum samples. It has also been used to dynamically monitor the excretion of PCN by *Pseudomonas aeruginosa* during its growth. The presence of PCN in 15 clinical sputum samples was detected by SERS, which indicates *Pseudomonas aeruginosa* infection, with 95.6% sensitivity and 93.3% specificity. The endotoxin detection method for PCN offers the benefits of quick detection, effective interference rejection, and in-place, real-time detection. The endotoxin detection limit was as low as 6.125 ng/mL, and the whole detection time was reduced to roughly 100 s [96]. In Gram-negative bacteria, lipopolysaccharides exhibit distinctive SERS spectral peaks, which are released during infection, and could be further analyzed by PCA. In the study, LPSs from *E. coli*, *S. typhimurium*, *S. minnesota*, *V. cholerae*, *Rhizobium species R. CE3*, and *R. NGR*, *Neisseria meningitidis*, produced unique spectra [97]. It has been demonstrated that the detection of bacterial endotoxin lipopolysaccharides can achieve a limit of detection (LOD) as low as 0.0003 endotoxin units (EU)/mL and 1 colony-forming unit (CFU)/mL using signal processing-based enhancement technique with a processing time less than one minute [85]. Figure 3 shows the process of SERS spectra collection.

Flavivirus-caused life-threatening diseases can also be identified using the biomarker non-structural protein–1 (NS-1), the antigen found in the febrile stage from serum samples. A bioconjugated gold nanoparticle-based SERS probe was used for the accurate detection of mosquito-borne flaviviruses [98]. Mouse IgG monoclonal antiflaviviral antibodies conjugated to the AuNPs-based SERS probe can be used as a sensitive fingerprinting detection tool for Dengue virus and West Nile virus. A detection limit of as low as 10 PFU/mL of Dengue virus and West Nile virus can be achieved, which is comparable with the sensitivity of quantitative PCR-based assays. Au substrates are used for SERS detection, followed by classification with SVM and compared for accuracy, specificity, and sensitivity [99]. 

### 3.3. Bacterial Whole Cell Detection

The vibrational spectroscopic technique of spontaneous Raman is used to identify the bacteria based on the Raman scattering spectra for cellular components, which enables rapid analysis of the samples. The spontaneous Raman technique is inherently weak compared to the elastic/Rayleigh scattering. Though the method requires a higher concentration of bacteria to be detected, the samples can be processed without any manipulations. The technique finds applications where sensitivity is not crucial but provides real-time analysis, whereas SERS enhances the signals and provides a susceptible platform for detection. Spontaneous Raman finds applications for bacterial identification directly from cultures on agar, such as Mueller–Hinton agar plates for *Staphylococcus* strains. They studied using 277 strains, which were cultured for 24 h and subjected to a 785 nm laser; the data were analyzed using PCA, LDA, and SVM, showing high accuracy. Therefore, the study enables the spontaneous Raman technique to be used as a diagnostic technique [100]. Due to the inherent weakness of spontaneous Raman signals from bacterial species and the low signal-to-noise ratio, it is difficult to identify species accurately, but the current advancements in machine learning enable precise detection [101,102]. Clinical samples have lower concentrations of bacteria and mixed species populations, which requires SERS as it helps overcome the weak Raman scattering. Machine learning techniques have shown higher accuracy in detecting spectra of resistant and sensitive strains of *Staphylococcus aureus*, with 89.1% accuracy with spontaneous Raman [101,103]. However, there are a few issues with spontaneous Raman; the number of Raman signals is only 10^−8^ of the incident photon, which is a weak signal. The stronger fluorescence from the background can hinder the sample spectrum, and the longer exposure to the laser can affect the sample. Hence, SERS helps to overcome the shortcomings of acquisition time and provides higher sensitivity to the Raman scattering.

Most studies provided evidence that bacteria’s SERS signals originate from their outer envelopes [104]. However, studies investigated the bacterium *Acidithiobacillus ferrooxidans* and reported that the SERS spectra displayed physical and chemical variations, which are even caused by different growth media [105]. The origins and contributions of the bacterial SERS signals were investigated to distinguish the cell membrane components of several bacteria. For example, hierarchy cluster analysis can distinguish the three strains of *Escherichia coli* and one strain of *Staphylococcus epidermidis* [106]. SERS could even identify the significant component of cell surface domains of *Shewanella oneidensis* MR1, which has the redox heme protein [104]. The features observed in bacterial SERS spectra depict the bacterial surface, with a few from the sample preparation stages having metabolic activity or molecular species detached from the bacterial surface [107]. Depending on the sample preparation with the colloids, the analysis of silver-treated bacteria showed intense and highly specific SERS spectra associated with many signals of flavin adenine dinucleotide (FAD), DNA, carboxylates, and phosphates. Silver-treated bacteria have shown that their SERS spectra were dominated by FAD, which is in the cell plasma membrane [88]. A higher lipid content of unsaturated fatty acids in the outer membranes of marine bacteria could also be identified by a comparative SERS study of psychrophiles, arctic marine bacteria, and common mesophilic bacteria [42]. 

**Figure 4:**
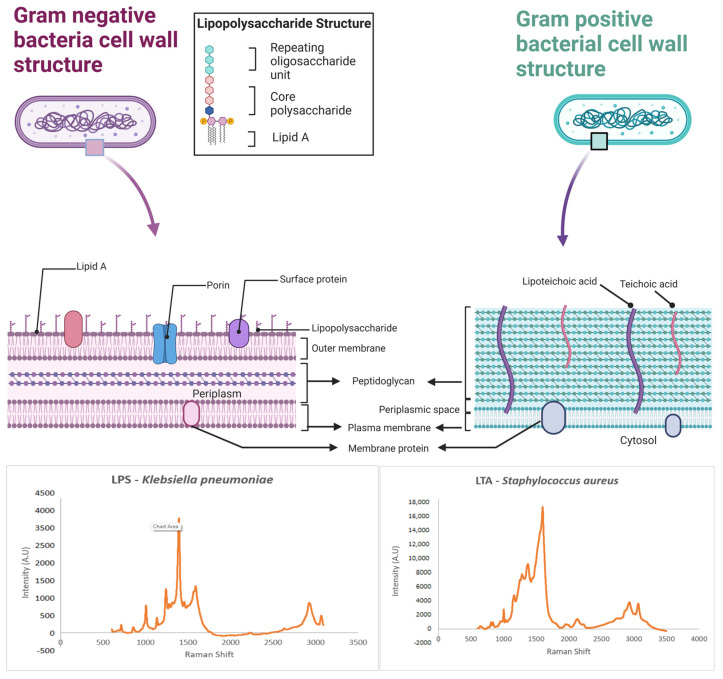
The cellular structure differences between Gram-positive and Gram-negative bacteria, SERS spectra of Gram-negative lipopolysaccharide and Gram-positive lipoteichoic acid. Created with BioRender.com (accessed on 19 July 2024).

If the bacterial SERS spectra originate mainly from the cell outer envelope, significant differences between Gram-positive (G+) and Gram-negative (G−) bacteria would be observed because the major distinction between these two kinds of bacteria lies in the external structure of their cell [78,108]. G− bacteria have an outer membrane and an additional periplasmic space within the outer membrane, which are both absent in the G+ bacteria. The G+ bacterial cell wall depicts multiple layers, having the peptidoglycan along with the teichoic and lipoteichoic acids and complex polysaccharides (Figure 4). These structural differences [108] would introduce spectral differences between G+ and G− bacteria [109]. The SERS spectra of *Staphylococcus aureus* (G+), *Klebsiella pneumoniae* (G−), and *Mycobacterium smegmatis* were successfully differentiated based on these bacteria’s outer structural differences. Figure 4 displays the cellular structure differences between G+ and G− bacteria and the SERS spectra of LPS for Gram-negative *Klebsiella pneumoniae* and LTA for Gram-positive *Staphylococcus aureus.*

Identifying unique SERS spectra depends on the Raman wave shift, where each peak is assigned to a corresponding structural element of the identified molecules. The list of chemical substances that could potentially contribute to SERS spectra is mainly from the outer structure of the bacterial cell, such as the outer membrane and cell wall. However, some of the inner cell components, such as DNA, RNA, proteins, and some metabolic products, such as the oxidized form of nicotinamide adenine dinucleotide phosphate (NADP^+^), nicotinamide adenine dinucleotide (NAD^+^), and adenosine triphosphate (ATP), may leak out of the bacterial cell to the substrate and then yield SERS responses. Chemical structures from both Gram-positive and Gram-negative bacteria are summarized in Table 2. SERS peaks from these components reported in the literature are summarized in Table 3, along with their possible assignments, which would provide some insights for assigning peaks and understanding the origins of SERS peaks.

## 4. Enhancing SERS Detection Performance

### 4.1. Different Types of SERS Substrates with Enhanced Sensitivity

As a valid and reliable detection platform, SERS-based biosensors need to be sensitive due to the low concentration of pathogenic bacteria in clinical and food samples. The SERS detection sensitivity of bacteria is mainly determined by SERS-active substrates. Different types of substrates have been fabricated to facilitate sensitive detection of bacteria, including silver metal deposits [22], silver colloid [9,26,53,116], gold colloid solutions [117], electrochemically roughened metal surfaces [118], silver film over nanosphere (AgFON) [119], silver nanorod (AgNR) array substrates [63,120], silver nanocrystal-assembled silver nanospheres (AgNSs) [65,120], silver-coated silicon nanowire arrays [119,121], and an array of Ag nanoparticles embedded in anodic aluminum oxide (AAO) nanochannel substrates [122]. These nanostructures have been proven to have an enhancement factor (EF) from 10^7^ to 10^10^ for small molecules [123,124]. 

For the detection of bacteria, one key factor is the enormous size of the bacterium (usually has a diameter of 0.5 μm and length of 1 μm) compared to the nanostructure (tens to one hundred nm), which limits the contact and the distance between bacterium and nanostructures [125]. The SERS principle indicates that the enhancement factor of SERS active substrates can be increased by reducing the distance between bacteria and nanostructures [126]. The distance can be minimized by physical force and chemical bonds. For example, the antibiotic vancomycin was used to entrap bacteria as it can bind nonspecifically to the cell wall [119,127]. The microscopic images of antibiotic-modified substrates with bacteria showed a distorted cell edge, depicting the closer bond to the substrate and enhancing the SERS effect 1000 times on lessening the distance [120]. The substrates are chemically assembled using a method of production that uses electrohydrodynamic flow to generate chemical cross-linking between colloidal gold nanospheres, resulting in optically uniform SERS substrates. The produced substrates display SERS signals across an area of 100 × 100 µm^2^ with a relative standard deviation of 10.4%. Molecular signal amplification is required in molecular diagnostics when the target molecule is identified due to the low signal-to-noise ratio. The effectiveness of the Au nanostar dimer structures as a substrate for SERS for the ultrasensitive and label-free detection of the pyocyanin molecule has been established with a detection limit of 335 pM [128]. The bioscaffold with integrated array microchamber nanostructures was prepared; the quantitative test method was applied for the in-situ detection of endotoxin released in the mixture of *P. aeruginosa* and bacteriostats [95]. A label-free and high-speed detection of *Staphylococcus aureus*, *Klebsiella pneumoniae*, and *Mycobacterium smegmatis* bacteria using silver on anodic aluminum oxide nanoparticle arrays as SERS substrates was reported, which would represent a novel approach for microbial diagnostics and biosensing [129].

### 4.2. Bacterial Concentration Methods

Combining SERS with pre-concentration can aid in sensitivity, which can capture as many microbes as possible in a fixed surface area with a large sample volume, such as magnetic field, capillary action, and optical tweezers [78]. For instance, magnetic-plasmonic nanoparticles were fabricated and used to enrich bacteria by applying an external magnetic field. Based on this strategy, *Escherichia coli K12*, *Pseudomonas aeruginosa*, and *Acinetobacter calcoaceticus* were detected with an LOD of 2 × 10^5^ CFU/mL [73]. A simple two-step filtration method that concentrates the bacteria up to 1000-fold before SERS measurements can also be used with a recovery rate of 79.1% [120]. A liquid core photonic crystal fiber (LCPCF), which is filled with the bacterial solution due to capillary force, exhibited a limit of detection at 10^6^ cells/mL of live bacterial cells of *Shewanella oneidensis MR-1* was achieved in an aqueous solution [46]. The detection of spores plays a pivotal role in early detection, as the combined optical trapping of bacteria using optical tweezers with SERS could capture and measure the single bacterial spore. Strain discrimination of *Bacillus stearothermophilus* spores was also achieved [21]. A label-free SERS detection platform for *Bacillus anthracis* spores using aptamer as capture, 10^4^ CFU/mL spores spiked in orange juice were successfully detected and discriminated between spores of *Bacillus anthracis* and *Bacillus mycoides* within 40 min [130].

One of the most common methods to separate and concentrate bacteria from complicated samples is immunomagnetic separation (IMS), which captures the bacteria from the sample matrix using an antibody-antigen reaction and separates the complex with magnetic force [121]. This strategy often includes magnetic nanoparticles that have been linked to antibodies or aptamers, which are specific to the bacteria. After the magnetic nanoparticles capture the bacteria, the applied magnetic field will separate them from the sample matrix [31]. To simultaneously detect multiple bacteria using a SERS aptasensor, the aptamer for *Salmonella typhimurium* and the antibodies of *Staphylococcus aureus* and *Escherichia coli* O157:H7 were immobilized on the surface of gold, silver, and silver core–shell nanoparticles, and then Raman dye molecules were marked on them. The mixture was filtered to detect the Raman signal. The results showed that the multiple bacteria in the mixture could be distinguished simultaneously with a detection limit of 100 CFU/mL. Therefore, the studies have shown the high sensitivity exhibited by different substrates [131].

### 4.3. Microfluidic SERS-Based Detection

Microfluidics integrates engineering, physics, chemistry, biochemistry, nanotechnology, and biotechnology to precisely control and manipulate the behavior of fluids that are geometrically constrained to a small scale, typically submillimeter. Since bacteria are often detected within solution, and the SERS-active substrates are generally in a small scale, it is natural to combine microfluidics with SERS to develop such lab-on-chip devices for bacterial detection. A microfluidics chip coupled with SERS to rapidly detect and differentiate methicillin-sensitive *S. aureus* (MSSA) and methicillin-resistant *S. aureus* (MRSA) from clinical isolates obtained from the United States and China. SERS was used to effectively identify and distinguish between 21 MSSA isolates and 37 MRSA isolates that were isolated from infected individuals, and the results were verified by using PCR and multilocus sequence typing (MLST) [75,132]. A SERS microfluidic device to quickly distinguish nine strains of *Escherichia coli* was developed [40], and a nanobiosensor chip for detecting *Escherichia coli* bacteria down to the concentration level of a single bacterium has been reported [133]. The chips used are highly enhanced plasmonic nanosculptured thin films of silver on a silicon platform, which enhances the Raman bands of adsorbed 4-aminothiophenol molecules. T-4 bacteriophages were immobilized on the previously mentioned chip for the specific capture of target *Escherichia coli* bacteria.

SERS in conjunction with microfluidic devices having micro/nano-filter membranes or integrated microchannels functionalized with vertically aligned Au/Ag-coated carbon nanotubes, were used to detect viruses [134]. Plasmonic Au-NPs help to further improve the Raman spectra. These tools may be able to effectively capture viruses from a variety of bodily fluids/secretions, such as saliva, nasopharyngeal secretions, tears, etc. Thus, they can raise the viral titer and make it possible to correctly identify viruses based on their own Raman signatures. It will enable quick screening of COVID-19 in both symptomatic and asymptomatic cases [135].

Clinical Raman spectroscopy translation was proven to be greatly enhanced by the combination of microfluidics and bioprinting platforms with SERS. As complex and effective as mixing, microfluidic systems make separation, parallelization, and multiplexing possible. Hence, they can lessen the quantity of sample preparation processes needed, lower the volume of samples and reagents needed, enhance sample uniformity, and boost throughput. Additionally, the development of label-free SERS substrates and data processing algorithms can enhance spectral signal and interpretability, which is critical for wide pathogen screening assays, and the creation of microfluidic and bioprinting platforms is achieved for speedy clinical sample processing [136]. Biosensors can serve as the cornerstone of quick point-of-care devices with the potential to improve patient care when they are coupled with cutting-edge microfluidic technologies.

### 4.4. Differentiation of Spectra Using Chemometric Analysis

A SERS-based bacterial detection platform must have the capacity to differentiate bacteria between species, strains, and even serotypes, which is due to the variety of bacterial pathogens present in real samples. Such differentiation can be based on the SERS response of the bacterial whole cell, DNA, or even biomarkers [137]. The strains *Mycobacterium indicus pranii* (MIP) and *Mycobacterium intracellulare* are two examples of those that have the same 16S rRNA sequence. MIP is significant since it has strong anticancer action and is utilized as an adjuvant for protection against leprosy and tuberculosis (TB). The opportunistic pathogen *M. intracellulare*, on the other hand, causes severe respiratory infections in AIDS patients. Given that these two bacterial species coexist in immunocompromised people, it is crucial to distinguish between them. Using multivariate statistical techniques like Raman and resonance Raman spectroscopy, it is possible to clearly discriminate between these two closely related bacterial strains. To demonstrate that MIP is biochemically different from *M. intracellulare*, differences in the mycolic acid profile and carotenoid pigments were found. Both MIP and *M. intracellulare* generated carotenoids; however, the latter produced more of them, according to resonance Raman tests [138]. 

To examine the metabolic processes of bacterial persister cells, coupled single-cell Raman imaging spectroscopy with deuterium oxide labeling was performed. Deuterium is taken up by metabolically active cells, which produce unique Raman bands as a direct indication of metabolic activity. The metabolic activity of *Mycobacterium smegmatis*, a rapidly proliferating model for Mycobacterium TB, using this imaging technique discovered that *M. smegmatis* persister cells exhibit specific metabolic activities and active cell development when the antibiotic rifampicin is present. Hence, the mechanism of persistence varies depending on the kind of bacteria and antibiotics utilized [139]. Utilizing Ag-coated silicon nanopillar SERS substrates, the biomarker of *Mycobacterium tuberculosis*, the SERS spectra of three primary types of mycolic acid (MA)—alpha MA, methoxy MA, and keto-MA—that together make up the entire amount of MA found in mycobacteria were effectively detected. The MA generated from three separate sources underwent label-free characterization. The SERS spectra of different types of MA from delipidated, undilapidated, and gamma-irradiated MA can differentiate between pathogenic and non-pathogenic forms. Hence, tuberculosis (TB) could be potentially directly detected from sputum samples [140]. However, in the presence of multiple bacteria in a sample, such as different serotypes and strains, the SERS signal of bacteria can be used to differentiate them among species, strains, and serotypes when the chemometric analysis is applied. Such chemometric analysis is proven to be limited when a mixture of different bacteria is present. In the presence of multiple bacteria, spectral features exhibited will depict an individual bacterium as it reports the vibrational modes of chemical bonds. Using mathematical modeling methods can reveal the individual spectra of the bacteria by differentiating [41]. However, such separation of the spectra can only be achieved under the circumstances that (1) the SERS responses of the individual bacteria retain the same intensity level and (2) the concentrations of the individual bacteria are close. If one of the two conditions is not satisfied, it will result in one bacterium SERS signal dominating the mixture spectra and masking the SERS signal of the others, which makes differentiation impossible. 

Immune-conjugated nanoparticles are the central component of immune SERS, which are unique to the target bacterium. The immune molecules are also labeled with reporters called Raman reporter molecules, exhibiting a strong and specific Raman spectrum. Hence, by identifying Raman reporter molecules, bacteria can be distinguished. Using antibodies, *Escherichia coli* [31,141] and *Staphylococcus aureus* [142,143] were successfully detected, and multiplexing was achieved as well [144]. Aptamer can also be used in conjunction with SERS for specific bacterial detection and differentiation. Multiplexing detection of bacteria using aptamers, such as [130] the anti-*Salmonella typhimurium* aptamers, anti-*Staphylococcus aureus*, and anti-*Escherichia coli* O157:H7 antibodies, were functionalized onto the Ag–Au core–shell nanoparticles labeled with unique Raman reporter molecules. Specific detection and differentiation between species (*Escherichia coli* O157:H7 vs. *Salmonella typhimurium*) and strains (*Escherichia coli* O157:H7 vs. *Escherichia coli K12*) were achieved as low as 10^2^ and 10^3^ CFU/mL under 45 min of total detection time. Therefore, secondary confirmation is mainly based on the target bacteria’s immunological properties and immune molecules, with the use of antibodies and aptamers.

SERS signal of bacteria originates from the cell wall and proteins [88], which causes visualization of differences in the spectra to be very difficult due to the similar chemical structures of different bacterial cell walls [127]. Thus, chemometric analysis is commonly used to enhance pattern recognition and facilitate species classification. It can also aid the model calibration of the SERS spectra of bacteria with a variety of statistical formats, including principal component analysis (PCA), hierarchical cluster analysis (HCA), partial least square discriminant analysis (PLS-DA), partial least square regression (PLS), discriminant function analysis (DFA), linear discriminant analysis (LDA), and support vector machine (SVM). On the contrary, a supervised method such as PLS-DA can also be used, where previous knowledge of the classes of the bacteria is used to yield sharper discrimination [120,145]. PLS-DA projects the data to a new set of coordinates, providing a positive/negative prediction by using a linear combination of the predictor variables. It is frequently used to differentiate bacteria based on well-known traits, such as Gram stain results, that is, Gram-positive versus G-. Other methods can also be used, and based on the study goals and the nature of the data, the appropriate chemometric methods can be chosen. Several publications have used PCA, HCA, LDA, etc., with many different bacteria up to 8 species, and such differentiations are easily possible as the structural variation of species is notable [9,35,76,146,147,148]. For example, 27 different bacteria isolated from 12 species were analyzed using SERS spectra recorded from vancomycin-functionalized (VAN) AgNR substrates. Researchers also explored the discrimination at subspecies levels up to four serotypes of *Salmonella* [120] and 14 strains of *Arthrobacter* [149]. The biofilm formation can also be identified as early as 3 h after inoculation with partial least squares regression. Thus, it can be further developed for quorum sensing using SERS substrates and machine learning and integrated into microfluidics [150].

Gram-positive *Staphylococcus aureus* and *Enterococcus* sp., as well as Gram-negative Escherichia coli, Klebsiella pneumoniae, Proteus mirabilis, Pseudomonas aeruginosa, and Enterobacter cloacae, were used to identify the many microbes responsible for urinary tract infections. After growth on the culture medium, a fiber Raman probe and a Raman spectrometer (830 nm) were used for Raman measurements. Colonies were removed from the agar surface and put on an aluminum foil. After preprocessing, PCA and Mahalanobis distance (PCA/MD) discriminating techniques were used on the spectra. The mean Raman spectra of several bacterial species display comparable bands, and strong bands associated with carotenoids served as a good way to identify *S. aureus*. PCA/MD, which could distinguish Gram-positive bacteria with 100% sensitivity and specificity [151]. The O-antigen, or bacterial fingerprint, establishes the specificity of the bacterial serotype. Molecular moieties in complex systems, like infections, can be identified from one another using molecular fingerprints detected in vibrational spectra. In addition, the excellent sensitivity and specificity attained by SERS make the benefits of vibrational Raman scattering exceptional. The primary spectroscopic distinctions between the O-antigens of *E. coli* O16 and *S. typhimurium* are provided by distinctive fundamental vibrational modes connected to the monosaccharide N-acetylglucosamine and deformations of the O-antigen chains. The indirect detection of Escherichia coli O16 and Salmonella typhimurium by extracting, purifying, and characterizing the O-antigen using silver nanoparticles has also been reported [66]. Fungal strains such as the human cryptococcosis can also be detected, such as the human cryptococcosis, which is most frequently brought on by the two species of *Cryptococcus neoformans* and *Cryptococcus gattii*. The identification of Cryptococcal infections is challenging due to the lengthy detection cycle of Cryptococcus in clinical specimens. The study directly differentiated between *C. neoformans* and *C. gattii* in clinical specimens using SERS and spectrum analysis using positively charged silver nanoparticles (AgNPs) as a substrate. The AgNPs self-assembled on the fungal cell wall’s surface through electrostatic aggregation. Using PCA, the innovative SERS detection approach can clearly discriminate between the Cryptococcus species [152]. Table 4 summarizes the literature on the analysis used for differentiating bacteria based on their SERS spectra.

### 4.5. AI/ML-Enabled SERS Detection 

In order to create links between spectral data and genetic variations, machine-learning approaches might potentially be used. For instance, they might be used to track the genetic change and evolution of bacteria and viruses or to research the real-time interactions between diseases and medications (antivirals or antibiotics). The creation of novel antivirals and antibiotics, as well as a better knowledge of the interactions between cells and drugs, is aided by comparing the spectrum variations in the Raman bands for proteins, nucleic acids, lipids, carbohydrates, and cholesterol. Finally, even in the absence of pharmacological additives, machine learning may be utilized to forecast drug susceptibility and the lowest inhibitory concentration [136]. The advancements in artificial intelligence combined with SERS have enabled the development of rapid detection. The principal component analysis (PCA) method has limitations due to the overlapped spectra and the presence of multiple bacteria in different kinds of media; hence, with machine learning tools, it shows better class separability and higher accuracy. The deep neural network called DualWKNet could differentiate *E. coli* and *S. epidermidis* from different media like water, artificial urine, ground beef solution, milk, and nutrient broth with a high accuracy of 98%. The DualWKNet, with a lesser number of convolutional layers but a larger kernel size, could consider the spectral features and differentiate the species with higher accuracy than PCA and logistic regression [154]. Identification of MRSA and MSSA spectra could not be classified with the naked eye, but peak intensity ratio differences and classification algorithms such as k-nearest neighbors (k-NN), support vector machine (SVM), decision tree (DT), and naïve Bayes (NB) can be used for differentiation [99]. With antimicrobial resistance being a rising concern with the label-free SERS spectra of MRSA and MSSA appearing similar and the AgNR exhibiting a higher signal-to-noise ratio. Hence, to differentiate with the use of a stack autoencoder (SAE)-based deep neural network, where the SERS peak difference was from the peptidoglycan layer. The performance of SAE-DNN was also compared with other classifiers like SVM and KNN, which show higher accuracy from raw data without preprocessing. This can also be used for rapid clinical detection of AMR [155]. 

A convolutional neural network (CNN) [156] trained on datasets of SERS was used to identify clinical samples, enabling better treatment outcomes. It fastens the process for accurate detection without conventional culture techniques. The training reference dataset was of 30 bacterial species and yeast; the binary CNN could differentiate between MRSA and MSSA with an accuracy of 89.1 ± 0.1%. The clinical samples were also used to refine the detection accuracy, which, in turn, increased the accuracy by 99.0 ± 1.9% for 50 samples [157]. A novel CNN, RamanNet, for the sole classification of SERS, has been developed for endotoxin detection. The algorithm was trained with spectral data of 11 bacterial LPS and has an accuracy rate of 100%. It is a densely connected form of neural layer network, using the triplet loss feature, which helps in reducing the dimensionality of data [80]. Bacterial identification using CNN and Siamese networks was compared; Siamese networks are made of a pair of CNN, and various other machine learning tools like PCA-LDA, PCA-SVM, PLS-DA, and PCA-RF were compared. The networks were assessed on their sensitivity and other parameters like predictability and time for training. Siamese model showed the highest level of sensitivity for bacterial detection [158]. The 16 different sequence types of *Klebsiella pneumoniae* could be differentiated by SVM, which was in correlation with multilocus sequence typing [47]. The carbapenem-sensitive and resistant strains of *K. pneumoniae* could also be differentiated by using CNN [159].

The SERS spectral recognition feature is another novel machine learning approach like facial recognition, wherein it matches the database. The characteristic peak similarity (CaPSim) method was used for spectra from three different substrates of AgNR, AuNP, and Au NS, considering the variability of data. Hence, it has shown high accuracy in the detection of chemicals [160]. Clinical translation is slowed by various factors such as issues with spectral enhancement consistency, difficulty in interpretation of spectrum, inadequate specificity, sensitivity, and an inefficient procedure from patient sample collection to spectral capture. To overcome these shortcomings, new capture and affinity agents, such as aptamers and polymers, and artificial intelligence will indicate the specific pathogens presence or absence. The creation of a database of the standardized spectrum can help to increase reproducibility and find applications in microbiological laboratories [156]. Therefore, the advent of machine learning has enabled the clinical translation of the SERS technique for culture-free detection of microbes and complex samples.

#### Reproducibility of SERS

One of the significant concerns related to the practicability of SERS has been the reproducibility of the data for various reasons, such as the different instruments in use. The instruments can show variability in results due to the equipment’s power, acquisition time, and calibration with standards. The samples need to be prepared using a similar protocol of incubation and centrifugation to reduce variability. The substrate fabrication can affect the variability; therefore, the uniformity of the monolayers needs to be maintained [101,102]. The AI-based data analysis can help as a database to reduce variability, such as RamanNet, which has been developed solely for SERS. RamanNet has been used with five different instruments to analyze the results, showing high accuracy (unpublished). It helps evade the critical issue of data reproducibility by measuring various bacterial biomarkers and reducing variability [79]. Therefore, with AI, SERS can mitigate the problem of reproducibility to a certain extent to be applied in clinical diagnostics. In general, SERS-based detection of bacteria has improved in the past decade. In the future, this sensitive method will be promising for detecting single bacteria in the real-world matrix by constructing SERS substrates with high activity or combining different techniques.

### 4.6. Detection of Microbes in Complex Samples

For the SERS-based detection platform to have a major impact in the field of detection of bacteria, the developed biosensors must detect bacteria in real samples, such as food samples [149] and clinical samples [127]. Studies of the detection of bacteria using SERS in real food samples are limited due to the complicated nature of the food matrix, which contains molecules like target pathogenic bacteria [65]. To solve the problem, a separation and detection method of many pathogens in food matrices by silica-coated magnetic probes (MNPs@SiO_2_) [121]. A sandwich assay was used to capture the target microorganisms directly from a food matrix, and these probes were functionalized with pathogen antibodies. Subsequently, AuNPs in combination with Raman reporter are also functionalized with particular antibodies. These probes with specific pathogen antibodies can capture the target bacteria directly from a food matrix, and the sandwich assay was formed using AuNPs with a Raman reporter functionalized with corresponding antibodies. With this assay, *Salmonella enterica* serovar Typhimurium and *Staphylococcus aureus* were detected in spinach solution and peanut butter as low as 10^3^ CFU/mL. These species are found to have infective doses varying from 10 to 10^8^, wherein the sensitivity of the assay can be increased by filtration of the sample before analysis. A label-free SERS-based bacteria detection in real food samples was reported [120] with VAN AgNR substrates used to directly detect pathogenic bacteria from mung bean sprout samples after a simple two-step filtration procedure. With this method, 100 CFU/mL of bacteria were identified from mung bean sprout samples, and differentiation between bacterial species and serotypes was achieved. One of the most dangerous pathogenic bacteria associated with foodborne illnesses is *Escherichia coli* O157:H7. SERS-based lateral flow immunoassay (LFIA) is a rapid detection biosensor for the sensitive and quantitative measurement of *E*. *coli* O157:H7 in biological samples. High-performance tags for the LFIA system based on SERS were created using a new monodispersed gold-shell silica-core (SiO_2_/Au) nanosphere (NP), with exceptional stability and great SERS activity. On the test lines, the SiO_2_/Au SERS tags that were modified with two layers of Raman reporter molecules and monoclonal antibodies successfully attach to *E*. *coli* O157:H7 and form sandwich immune complexes. Observing the test lines Raman intensities, *E. coli* O157:H7 was quickly and quantitatively identified. Such applications can be utilized in the detection of biological samples like tap water, milk, human urine, and lettuce extract of biological samples like tap water, milk, human urine, lettuce extract, and beef [161]. The foodborne pathogens can be detected by SERS on AgNR substrates by organisms such as *Salmonella*, *Staphylococcus aureus*, and *Escherichia coli* O157:H7. A silver colloidal nanoparticle suspension along with the cells could be detected at 785 nm. The assay exhibited a rapid and very sensitive detection, followed by spectral data reduction of PCA and HCA to distinguish between species [162]. A particular single-stranded DNA aptamer is utilized to specifically capture and label *Salmonella enteritidis*. The modification with an extra adenine and fluorescein (FAM) was employed as an indicator for the presence/absence of *Salmonella enteritidis*. The aptamer signals were obtained by further filtering of gold nanoparticles, and they were then utilized to build a SERS mapping showing the presence and absence of target bacterial strains with possible quantitative capacity. This study shows the filtration-based SERS platform’s capacity to detect *Salmonella enteritidis* in a range of aqueous matrices, including distilled water and the water used to rinse fresh produce, with good selectivity and sensitivity [163].

Besides food and environmental samples, human/animal body fluid samples are one of the most important complexes, especially for clinical diagnostics. AgNR substrate-based SERS are used for the detection and differentiation of *Mycoplasma pneumoniae* in culture and in spiked and true clinical throat swab samples [145]. High sensitivity and specificity were reported with the assay, and a lower detection limit exceeded standard PCR. The use of vancomycin coated with anodic aluminum oxide substrates to directly detect bacteria in human blood samples has also been reported [127]. The bacteria capture property of vancomycin eliminates interference from human blood, thus producing a successful detection. A similar strategy was used to selectively capture bacteria from blood samples with vancomycin-coated silver–gold bimetallic SERS substrates [71]. *Escherichia coli*, *Salmonella enterica*, and *Staphylococcus epidermidis* are successfully detected in blood samples with SERS. For precise and speedy, label-free electrochemical detection of harmful food-borne bacteria like *Salmonella enterica*, a biosensor employing reduced graphene oxide-carbon nanotubes (rGO-CNT) nanocomposite has been developed. The nanocomposite was then cast onto the glassy carbon electrode and then further altered using DNA aptamers that had undergone amination. The resulting ssDNA/rGO-CNT/GCE aptasensor was then employed using the differential pulse voltammetry (DPV) approach to identify bacteria. With a limit of detection of 10^1^ CFU/mL under ideal experimental circumstances, the aptasensor could detect *S*. *typhimurium* throughout a large linear dynamic range from 10^1^ to 10^8^ CFU/mL [164]. Due to the quick onset and high fatality rates of bacterial meningitis, prompt identification of germs present in the cerebral spinal fluid (CSF) and subsequent efficient treatment are essential. A new quantitative assay to identify the three microorganisms that cause bacterial meningitis, combining SERS and lambda exonuclease, was reported. A processive enzyme called I-exonuclease breaks down one strand of double-stranded DNA that has a terminal 5-phosphate group. The novel assay format entails the simultaneous hybridization of two complementary DNA probes, one of which contains a SERS active dye, to a target sequence, followed by the digestion of double-stranded DNA with I-exonuclease and the detection of the digestion by SERS of the resultant material. In a multiplexed assay, three meningitis pathogens were effectively detected with predicted detection limits in the pico-molar range, providing quick results. This is the first paper to demonstrate that the quantification of each pathogen is achievable by combining partial least squares (PLS) regression with the distinctive spectral characteristics of the SERS signals [165].

Urinary tract infections (UTIs) are a major concern as over 60% of women develop a UTI during their lifetime, and it can cause nosocomial infections. The conventional culture method requires 24 h to obtain results using Raman-based methods to identify bacteria by single bacterial cell analysis and in suspension using dielectrophoretic forces. There was no hindrance caused by the antibiotics or any growth-inhibitory substances. The detection method in suspension requires the sample to be placed in the chip with four gold electrodes and can be identified from the established database. The detection of vancomycin-resistant and sensitive strains of enterococci using Raman-based methods can be identified rapidly based on the molecular changes caused by the drug action that affect the Raman spectra. Changes are seen just after 30 min when the drug concentration is above the minimum inhibitory concentration. The method could differentiate and identify *Enterococcus faecalis* strains as sensitive or resistant and can be applied to real-world samples [166]. The direct detection of UTI-related pathogens is performed without sample pretreatment (*Escherichia coli* CFT 073, *Pseudomonas aeruginosa* PAO1, and *Proteus mirabilis* PRM1) using SERS chips, which capture negatively charged bacteria [167]. Hence, as a diagnostic tool for UTI, Raman spectroscopy should be able to (1) determine whether urine sample results are positive or negative based on the bacterial burden for UTI, (2) identify the microorganism responsible for the positive samples, and (3) determine the antibiotic or antibiogram sensitivity of the implicated microorganisms. SERS could be further developed as a point-of-care device to detect UTIs [168].

## 5. Challenges and Opportunities

Due to technical limitations, the SERS-based detection of bacteria has faced increased challenges and opportunities. Earlier Ag NPs were used as the SERS substrates for detecting bacteria, which is easily affected by the external environment, thus resulting in weak reproducibility. As a result, common bacteria can be detected at the strain level and identified only at this period, such as *Escherichia coli*, *Pseudomonas*, *Staphylococcus*, and *Salmonella*. The differences in the SERS spectra of the bacteria were small, and the volume ratio of analyte to silver colloidal particles must be within a narrow range of values [110]. To broaden the species of bacteria detected by SERS, a variety of specific SERS-active substrates have been fabricated. Endotoxins, such as LPS, act as pollutants and affect Au NP. The investigation was contrasted with LPS-coated surfaces, coatings with tiny molecules (lipoic or citric acid), which show high binding, and bulk molecules (branched polyethylenimine-BPEI and polyethylene glycol-PEG), which demonstrated substantial repulsion to inhibit LPS binding [169]. 

Despite its excellent sensitivity, a problem for bacteria detection by SERS is how to achieve the higher detection sensitivity. Different pre-treatment strategies were combined with SERS, which improved the detection sensitivity. A facile synthesis of Au-coated magnetic nanoparticles as SERS substrate for the indirect detection of *Staphylococcus aureus* by detecting the reporter molecule, which showed an excellent detection limit of 10 cells/mL [59]. Rapid and sensitive detection of *Salmonella* showed a better calibration curve obtained in the range of 15 to 1.5 × 10^6^ CFU/mL [64] using the Au@Ag core/shell nanoparticles as SERS substrate. The most attractive aspect of bacterial detection is sensing bacteria in the real-world matrix. The magnetite-gold nanoparticles were used for *Escherichia coli* detection in apple juice [48], which shows an effective way of pre-concentration, separation, and detection of low levels of target pathogen (10^2^ CFU/mL) from liquid food matrix. Two different nanostructures, the Au@pNIPAM hydrogel with embedded Au nanorods and the mesostructured Au@TiO_2_ substrate with a mesoporous TiO_2_ thin film over a submonolayer of Au nanospheres, were used to detect *Pseudomonas aeruginosa* in vivo [55]. *Escherichia coli* and *Staphylococcus aureus* were detected in different fluids, such as blood, urine, water, or milk, using a polymer mat covering a layer of gold as a SERS substrate [50].

Label-free assays make it simple to prepare samples and provide quantitative real-time measurements, but they are susceptible to matrix effects and non-specific bindings. While the multistep methods for labeled assays make them slightly more difficult, the inclusion of numerous binding events boosts specificity, and amplification tags improve sensitivity. When combined, labeled assays seem to have a stronger chance of being translated into the clinic, especially when working with real samples. There are also challenges in integrating these components into a fully automated, standalone platform that can be used by end users without technical skills and a generalized integrated system that can handle a greater variety of clinical samples, including urine, blood, and saliva for various infectious viruses or bacteria [170].

Several analytes found in real-world samples, including bodily fluids, contaminants found in soil, and explosives, are disseminated in liquid, solid, or air phases. The development of a platform that can accurately and specifically identify these analytes in each of these stages is still a difficult task. The single-cell era of microbiology research has been made feasible by the union of Raman-activated cell sorting (RACS) and single-cell Raman spectra (SCRS) with distinctive fingerprint properties. Systems for Raman-activated Cell Sorting (RACS) are important tools for identifying the genotypes and phenotypes of certain bacteria [171].

The creation of sensitive and quick methods for viral detection has become crucial during the COVID-19 pandemic. A SARS-aptasensor based on colloidal solutions, which are rapid and specific to quantitatively determine the SARS-CoV-2 virus and distinguish it from other respiratory viruses, is one such method. For the separation of a truly straightforward and secure biofluid-like saliva from a current or past infection by SARS-CoV-2, an alternate Raman technique was recommended. The COVID-19 patient signal could be distinguished using the Raman-based classification model with accuracy, precision, sensitivity, and specificity of greater than 95% [172]. 

## 6. Conclusions

This review highlights the recent developments in microbial detection using SERS over the last decade, highlighting how these assays perform better in complicated samples in terms of sensitivity and specificity. These studies demonstrate that SERS-based detection methods could rapidly detect low concentrations of bacteria with high sensitivity as well as specificity. The differentiation between bacterial species, strains, and serotypes could be successfully achieved among various bacteria, either through chemometric analysis of the bacterial SERS signal or by using secondary confirmation. The synergistic use of microfluidics utilizes the advantages of SERS and subsequently develops numerous lab-on-chip devices. Although these exciting research results dramatically advanced the field compared with just a decade ago, the research on the practical use of SERS for real-world samples is limited. Perhaps the greatest barriers to a widely accepted SERS platform in the industry are the relatively prohibitive cost of SERS-active substrates, the complexity posed by the various biological and environmental samples, and the presence of multiple organisms in a sample. Nowadays, SERS is recognized as a valuable option for biological and chemical analytics, and with AI, it will be utilized as a powerful and reliable detection platform.

## Figures and Tables

**Table 1:** Summary of data on general SERS detection strategies for different bacteria.

Bacteria Strains	Detection Method	Substrates	Limit of Detection	Condition of Detection
*Escherichia coli*	Label-free detection	Silver nanoparticles	Down to single cell	Liquid (Lab test) [35,36,37]
			4.3 × 10^3^ cells/mL	Liquid (Lab test) [38]
			2.5 × 10^2^ cell/mL	Liquid (Lab test) [39]
			-	Liquid (Lab test) [23,40,41,42,43,44,45,46]
		Planar monolithic porous polymer layers functionalized with gold nanoparticles	-	Solid (Lab test) [47]
		magnetite–gold magnetic nanoparticles	10^2^ CFU/mL	Liquid (in apple juice) [48,49]
		Vancomycin-coated long-range ordered 3D nanoassembly of gold/silver core–shell nanorods with edge-on substrate	-	Solid (Lab test) [50]
		A polymer mat covered a layer of gold	-	Solid (in blood, urine, water or milk) [51]
	Label-based detection	Iron oxide-gold core–shell nanoovals; QSY21 as target	210 CFU/mL	Liquid (Lab test) [52]
		Citrate-stabilized gold nanosphere and hexadecyltrimethylammonium bromide (CTAB)-stabilized gold nanorod particles	2.0 × 10^2^ CFU/mL	Liquid (in water sample) [53]
*Pseudomonas*	Label-free detection	Silver nanoparticles	10^3^ CFU/mL	Liquid (Lab test) [54]
		Roughened metal shelter	10^6^ CFU/mL	Liquid (in diluted blood) [55]
		Au@pNIPAM hydrogel with embedded Au nanorods and mesostructured Au@TiO_2_ substrate with a mesoporous TiO_2_ thin film over a submonolayer of Au nanospheres	3.4 × 10^7^ CFU /mL	Liquid (in vivo) [56]
	Label-based detection	Silver nanorod array; pyocyanin as the biomarker	5 ppb; 2.38 × 10^−8^ mol/L	Solid (in clinical sputum samples: wounds and urine specimens) [57]
*Staphylococcus*	Label-free detection	Silver nanoparticles	Down to single-cell	Liquid (Lab test) [35,36]
		Silver nanoparticles	-	Liquid (in diluted blood) [58]
		Silicon wafer decorated with silver nanoparticles	10^2^ cells/mL	Solid (in human blood) [59]
	Label-based detection	Au-coated magnetic nanoparticles core/shell nanocomposites; DTNB as target	10 cells/mL	Liquid (Lab test) [60]
		GA-modified Au@Rubpy/L-GO SERS tags	-	Liquid (Lab test) [61]
		Gold nanoparticle-on-wire; DNAs as target	10 pmol/L	Liquid (Lab test) [62]
*Salmonella*	Label-free detection	Vancomycin-coated silver nanorod	100 CFU/mL	Solid (in fresh produce) [63]
		Silver nanoparticles	-	Liquid (Lab test) [43]
		Silver nanorod array substrates	Down to single cell	Solid (Lab test) [64]
		Au@Ag core/shell nanoparticles	15 CFU/mL	Liquid (Lab test) [65]
		Ag nanocrystals into Ag nanospheres	10 CFU/mL	Liquid (Lab test) [66]
*Salmonella*	Label-based detection	Silver nanoparticles; O-antigen as target	-	Liquid (Lab test) [67]
*Shewanella*	Label-free detection	Biofilms cultivated on gold-coated glass slides, gold nanoislands	-	Liquid (Lab test) [68]
		Tip-coated multimode fiber, liquid core photonic crystal fiber	10^6^ cells/mL	Liquid (Lab test) [69]
		Ag or Au colloidal particles onto a rigid, ceramic filter	-	Liquid (Lab test) [70]
	Label-based detection	Gold nanoislands; the intracellular bioreduction of two stable valence forms of chromate	Down to single cell	Liquid (Lab test) [71]
*Bacillus*	Label-free detection	Rough silver (colloidal) film	-	Liquid (Lab test) [72]
		Thin gold layer on an electrochemically roughened nanoscopic silver substrate	-	Solid (in human blood) [73]
	Label-based detection	AuNPs/PVP/Au; dipicolinic acid as a biomarker	~10^6^ (SERS EF)	Liquid (Lab test) [74]
		Fe_3_O_4_–Au core–shell nanoparticles	-	Liquid (Lab test) [74]
Other bacteria				
*Helicobacter pylori*	Label-free detection	Silver nanoparticles	~10^11^ (SERS EF)	Solid (Lab test) [75]
*Listeria monocytogenes*	Label-free detection	silver nanoparticles	Down to single cell	Liquid (Lab test) [35]
*Klebsiella*	Label-free detection	Vancomycin-coated silver nanorod	Bacterial strain level	Solid (Lab test) [75,76]
*Citrobacter*	Label-free detection	Vancomycin-coated silver nanorod	Bacterial strain level	Solid (Lab test) [75,76]
*Proteus*	Label-free detection	silver nanoparticles	Bacterial strain level	Liquid (Lab test) [9,77]
*Arthrobacter*	Label-free detection	silver nanoparticles	-	Liquid (in soil and groundwater) [78]
*Sphingomonas*	Label-free detection	silver nanoparticles	-	Liquid (in soil and groundwater) [78]
*Shigella sonnei*	Label-free detection	silver nanoparticles	-	Liquid (Lab test) [45]
*Mycobacterium smegmatis*	Label-free detection	Silver on anodic aluminum oxide nanoparticle arrays	-	Solid (Lab test) [29]
*Erwinia amylovara*	Label-free detection	silver nanoparticles	-	Liquid (Lab test) [45]
*Stenotrophomonas maltophilia*	Label-based detection	Gold nanoparticle-on-wire; DNAs as target	10 pmol/L	Liquid (Lab test) [61]
*Vibrio vulnificus*	Label-based detection	Gold nanoparticle-on-wire; DNAs as target	10 pmol/L	Liquid (Lab test) [61]

**Table 2:** The chemical structures of bacterial cells that may contribute to the SERS of bacteria.

Structure	Chemical Constituents	Gram
Cell wall		
Peptidoglycan [110]	Alternating polymers of NAM (*N-Acetylglucosamine*) and NAG (*N-acetylmuramic acid*)	+/−
Teichoic Acid [111]	Polyribitol phosphate or glycerol phosphate is cross-linked to peptidoglycan.	+
Lipoteichoic Acid [112]	Lipid-linked teichoic acid.	+
Periplasmic Space [112]	proteases, phosphatases, lipases, nucleases, and carbohydrate-degrading enzymes	−
Outer Membrane [112]	Phospholipids with saturated fatty acids.	−
Proteins [112]	Porins and lipoproteins transport proteins.	−
Lipopolysaccharide [112]	Lipid A and core polysaccharide	−
Other external structures		
Capsule [113]	Polysaccharides (disaccharides and trisaccharides) and polypeptides.	+/−
Pili [113]	Pilin and adhesins.	+/−
Flagellum [113]	Motor proteins, flagellin.	+/−
Biomarker Proteins [114,115]	For example, M proteins of streptococci and O antigen. Staphyloxanthin for *Staphylococcus* sp. Pyocyanin for *Pseudomonas* sp.	+/− + −
Other internal structures		
Metabolic products [108]	ATP, NAD, and NADP+	+/−
Proteins [9]	Metabolic proteins	+/−
DNA or RNA [9]	Nucleotides	+/−

+/−: Peptidoglycan layer is present in both Gram-positive and Gram-negative bacteria; however, it is much thicker in Gram-positive bacterial cells.

**Table 3:** SERS peaks from the components that contribute to the SERS of bacteria.

Chemicals	Peak Position (cm^−1^)	Tentative Peak Assignments	Chemicals	Peak Position (cm^−1^)	Tentative Peak Assignments
Cell wall			Other external structures		
Peptidoglycan (NAG) [110] SERS (514.5 nm)	699	N/A	Capsule	N/A	
	815	N/A	Pili	N/A	
	964	N/A	Flagellum [113] Raman (532 nm)	903	N/A
	1059	N/A		945	Skeletal CCN deformation
	1236	N/A			
	1279	N/A		1003	Phe
	1374	N/A		1246	Helix
	1394	N/A		1320	N/A
	1536	N/A		1453	CH_2_ rocking
	1638	N/A		1662	Amide I
Teichoic acid [111] Raman (532 nm)	964	POH bending	Other internal structures		
	1250	PO- bending	Cell plasma SERS [108] (514.5 nm)	735	N/A
	1212	CN bending		1330	N/A
	1322	CHOH bending		780	N/A
	1452	CH		1050	N/A
	1646	Amid II		1125	N/A
Lipoteichoic acid [111,112]	Similar to teichoic acid			1230	N/A
				1435	N/A
Periplasmic space [112]	N/A		Metabolic products (4-ATP) [108] SERS (632.8 nm)	1089	NH_2_ rocking
				1176	CH bending
Outer membrane proteins (Porins and OmpA) [112] Raman (514.5 nm)	1553	Trp		1211	CN bending
	1579	Trp		1286	CH stretching
	1602	Phe		1492	CC stretching and CH bending
	1613	Tyr			
	1669	Amide		1593	CC stretching and NH_2_ bend.
	1734	N/A			
Lipopolysaccharide [112] Raman (514.5 nm)	1612	N/A	Internal proteins [9] SERS-gold (830 nm)	1250	Amide III
	1652	N/A		1322	Adenine, guanine, and Tyr
	1726	N/A		1003	C(CC) aromatic ring (Phe)
N/A Not available				1081	V(PO) in oligonucleotides
			DNA/RNA [9] SERS-gold (830 nm)	546	CO and POC bending
				795	V(PO2) and v(CC) ring breathing
	
				816	CO and POC
				853	1,4 glysosidic link

**Table 4:** A literature summary on using chemometric analysis to differentiate bacteria based on SERS technique.

Chemometric Methods	SERS Substrates	Bacterial Samples	Number of Bacteria	Results and Conclusions
DFA-HCA; PCA [9]	Silver colloid	Clinical bacterial isolates from patients with UTI (*Escherichia coli*; *Klebsiella oxytoca*; *Klebsiella pneumoniae*; *Citrobacter freundii*; and *Enterococcus* spp. and *Proteus mirabilis*)	6 species, 5 strains	Discriminate between distinct species and discriminate *Escherichia coli* on strain level.
PCA, HCA, and DFA based on the “barcoding method” [146]	Au-nanoparticle-covered SiO_2_ substrate	*Bacillus thuringiensis; Bacillus cereus; Bacillus anthracis*; *Bacillus licheniformis*; *Mycobacterium smegmatis*; *Mycobacterium fortuitum*; *Escherichia coli*; *Salmonella typhimurium*	8 species	Species and strain separation
PCA, HCA, and PLS-DA [145]	AgNR	*Mycoplasma pneumonia* and clinical throat swab	1 specie, 3 strains	The throat swab samples spiked with *M. pneumonia*, and actual clinical throat swab samples were correctly classified.
PCA [35]	Internal deposition of silver nanoparticles	*Staphylococcus epidermidis* and *Escherichia coli* O157:H7	2 species	Differentiate *Staphylococcus. epidermidis*, *Escherichia coli* O157:H7, and their 1:1 ratio mixer
PCA [147]	Au, ion-doped SiO_2_ sol–gel	*Kembolar pneumonia*, *Escherichia coli*, *Pseudomonas aeruginosa*, *Enterococcus faecalis*, and *Staphylococcus aureus*	4 species, 2 strains	Discriminate SERS spectra of different bacteria and the culture media in which they are grown.
PCA and SVM [40]	Silver colloid incorporates a microfluidic device	*Escherichia coli*	9 strains	Classification between strains with a high correct rate
PCA [153]	Silver nanoparticles	*Enterococcus faecalis*; *Streptococcus pyogenes*; *Acinetobacter baumannii*; *Klebsiella pneumoniae*	4 species	Discrimination between G+ and G-bacterial genera
PCA, LDA, and HCA [149]	Roughened gold-coated glass slides	*Arthrobacter* strains	14 strains	Distinct molecular differences on the surface of fourteen closely related *Arthrobacter* strains; liquid and solid cultures are distinguished
PCA [73]	Magnetic–plasmonic Fe_3_O_4_–Au core–shell nanoparticles (Au-MNPs)	*Acinetobacter calcoaceticus*, *Escherichia coli K12*, and *Pseudomonas aeruginosa*	3 species	Discriminate between species
PCA and HCA [76]	Gold nanoparticles (GNPs)	*Salmonella typhimurium* ATCC 50013, *Salmonella* O7HZ10, *Shigella boydii* CMCC51514, *Shigella* sonnei CMCC51529, *Shigella dysenteriae* CMCC51252, *Citrobacter freundii* ATCC43864, and *Enterobacter sakazakii* 154	6 species, 2 strains	Discriminate between species and serotypes
PCA [63]	AgNR	Generic *Escherichia coli*; *Escherichia coli* O157:H7; *Staphylococcus aureus*; *Salmonella typhimurium* 1925-1 poultry isolate, and *Escherichia coli* DH 5a	3 species, 3 serotypes	Distinguish between distinct species, differentiate pure cell samples from mixed cell samples, and classify different bacterial strains.
PCA and PLS-DA [120]	VAN AgNR	*Salmonella enterica* serotype Anatum, *Salmonella enterica* serotype Cubana, *Salmonella enterica* serotype Stanley, *Salmonella* Enteritidis, *Escherichia coli* O157:H7, and *Staphylococcus epidermidis*	3 species, 4 serotypes	Differentiate between species and serotypes in mung bean sprout samples
PCA and machine learning algorithm—RamanNet [79]	AgNR	*E. coli*, *S. typhmirium*, *S. minnesota*, *S. mileloti*, *P. aeruginosa*, *M. catarrhalis*, *H. pylori GU2*, *F. tularensis LVS*, *E. coli 0128B12*, *E. coli 011B4*, *E. coli J5*, and *E. coli H100*	6 species, 7 strains	Discriminate between distinct species and discriminate on strain level
PCA [96]	AgNR	*E. coli*, *S. typhimurium*, *S. minnesota*, *V. cholerae*, *Rhizobium species R. CE3*, and *R. NGR*, as well as *Neisseria meningitidis*	6 species	SERS spectra can be used to differentiate between the different enteric LPS

## Data Availability

All cited references are listed in PubMed and Web of Science.
